# Management of Displaced Intra-Articular Calcaneal Fractures: A Comparative Study of Open and Minimally Invasive Surgery

**DOI:** 10.7759/cureus.9547

**Published:** 2020-08-04

**Authors:** Amirul Islam, Charles Mcdonald, Ahmed Aljawadi, Noman Niazi, Anand Pillai

**Affiliations:** 1 Trauma and Orthopaedics, Wythenshawe Hospital, Manchester, GBR; 2 Surgery, University of Manchester, Manchester, GBR

**Keywords:** displaced intra-articular calcaneal fracture, cost analysis, radiological outcome, sinus tarsi approach, minimally invasive surgery

## Abstract

Objectives

The ideal treatment of displaced intra-articular calcaneal fractures continues to be a subject of debate. The aim of the study was to compare the radiological outcome, cumulative radiation exposure, surgical time, time to surgery, wound healing times and cost involved in minimally invasive surgery (MIS) and open reduction internal fixation (ORIF) for calcaneal fractures.

Methods

This was a retrospective study of 39 calcaneum operated in our unit during 2012 to 2019, of which 20 had undergone ORIF and 19 had been operated upon following MIS.

Results

A total of 39 calcanea (37 patients) were operated, of which 20 had open procedure and 19 had MIS procedure, including one bilateral surgery in each group. Mean age of the patients in the MIS group was 42.18 years (range: 15-68 years) and that of the patients in the open group was 43 years (range: 21-75 years). Of the fractures, 53.84% (n = 21) was Sanders type III, 28.20% (n = 11) was type II and 17.94% (n = 7) was type IV. There was no statistically significant difference in the mean correction of Bohler’s angle and Gissane’s angle between the groups. The mean cost for implant used for each open procedure was £882.79, and the implant cost for each MIS procedure was £142.89. Mean utilisation of cumulative X-ray dose was significantly higher in MIS (0.764 mGy) in comparison to open surgery (0.392 mGy). The average surgical time for MIS was 64.9 minutes and that of open surgery was 106.3 minutes. Average waiting time for MIS was 6.6 days and that for ORIF was 9.8 days. Wound healing was quicker (average 13.4 days) in MIS than ORIF (average 17.2 days). All these differences were statistically significant.

Conclusions

Minimally invasive calcaneal fracture surgery is quicker and cheaper and can be performed earlier. It is associated with early wound healing, although it requires higher cumulative radiation dose.

## Introduction

Calcaneal fractures (CFs) account for more than 60% of tarsal bone fractures and around 2% of all fractures [1]. This can be a debilitating fracture and is most common in those who are economically active [2]. Ideal management of these fractures remains controversial. Open reduction internal fixation (ORIF) is usually performed utilising an extensile lateral approach, and plate and screws are used for internal fixation. However, this has been reported with 15% to 25% of wound-related complications or flap necrosis [3].

Current evidence suggests that less invasive surgical options could achieve equally satisfactory radiological outcomes compared to open techniques [3,4]. We have analysed pre- and post-operative radiological parameters such as Bohler’s angle, critical angle of Gissane, and calcaneal height and width. We have also investigated costs involved in the fixation method to find out any difference. In our study we also looked into the cumulative radiation exposure during fluoroscopy, time to surgery, duration of surgery, wound healing and other complications for both minimally invasive surgery (MIS) and open CFs.

## Materials and methods

A retrospective review was undertaken of all patients who underwent either MIS or ORIF for CFs in our unit from 2012 to 2019. The type of surgery was preference and practice of different foot and ankle (F&A) units of the same trust. Surgery was performed in all cases by a consultant F&A surgeon. A total of 37 patients were identified. Data were collected from a number of sources including electronic patient records, discharge summaries, clinic letters and Picture Archiving and Communication System (PACS).

Outcome measures included a number of key radiological markers such as Bohler’s angle, Gissane’s angle, and calcaneal width and height. Pre- and post-operative measurements were calculated to find out the amount of correction achieved and their statistical significance.

We also looked into the cost of implants involved in the fixation method and the cumulative radiation dose while using fluoroscopy during the procedure. Cost of implant was obtained from hospital procurement, and fluoroscopic dose was obtained from the PACS record. Surgical time was calculated from the Operating Room Management Information System (ORMIS) records.

**Figure 1:**
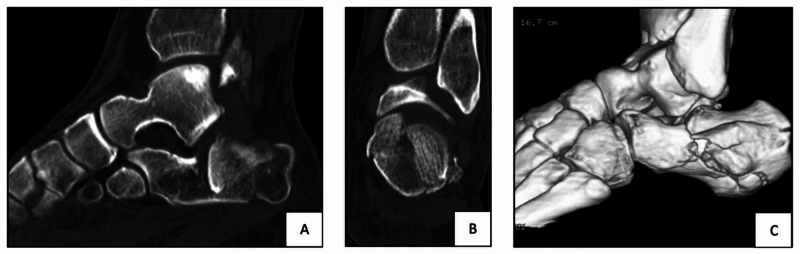
Sagittal (A), coronal (B) and three-dimensional reconstruction (C) CT scan of the calcaneal fracture treated by MIS fixation. *Same patient’s intra-operative photograph and fluoroscopic images are shown in Figures 2, 3, respectively. MIS, minimally invasive surgery

All patients had isolated injuries, and soft tissue status was carefully assessed. All patients had plain radiograph as well as computed tomography (CT) scan (Figure 1), and fractures were classified according to Sanders [5]. Initial management included below knee slab in a neutral position, leg elevation, analgesia and prophylaxis for venous thromboembolism. Surgery was performed once the soft tissue condition permitted as evident by the reduction of swelling and appearance of skin wrinkles.

Sanders classification [5] is based on the number of articular fragments seen on coronal CT scans at the widest point of the posterior facet of the calcaneum. It has been shown to be useful in determining treatment as well as prognosis [5].

**Figure 2:**
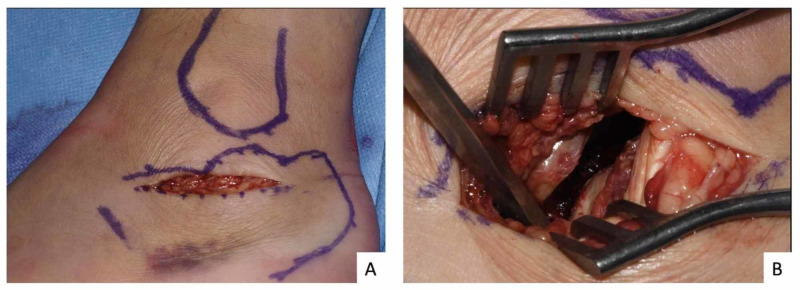
(A) Incision for MIS (limited sinus tarsi approach). (B) Restoration of the posterior facet using a small osteotome. MIS, minimally invasive surgery

All patients were operated in a lateral position under tourniquet control after receiving prophylactic intravenous antibiotics. For open surgery cohort (n = 20), extensile lateral approach was followed with meticulous tissue dissection to raise full-thickness flap. After open reduction, appropriate size locking plate (Zimmer Biomet A.L.P.S, Swindon, UK) was used for fixation. For the MIS cohort (n = 19), limited sinus tarsi approach was used (Figure 2). The displaced intra-articular fracture was reduced with the help of small osteotomes and elevators under fluoroscopic guidance and findings from the pre-operative study of CT scan. To hold the articular reduction, 4-mm cannulated screws were used. For extra-articular reduction, especially to get the calcaneum out to length and to correct the varus deformity, a 3.2-mm wire was used for joy-stick manoeuvre following the Essex Lopresti technique [6,7] and 6.5-mm cannulated screws were used for fixation of the tuberosity. Counter sinking was performed to avoid pressure over the skin and symptoms arising from this.

During the post-operative period, elevation of the operated limb was maintained along with nonweight-bearing mobilisation. Patients were discharged home after wound check on the second post-operative day with below-knee lightweight cast. Further wound check was performed in the clinic weekly until the wound healed completely, whereas cast was continued for a total of six to eight weeks. The first follow-up after surgery did not always take place on the same day of the following week because of the difference in trauma list and fracture clinic appointments. Additional nurse lead wound review was arranged to record wound healing. Radiological assessment was performed while patients came for the removal of their casts at six weeks and then at three months and six months. Patients were followed up till radiological union was confirmed.

**Figure 3:**
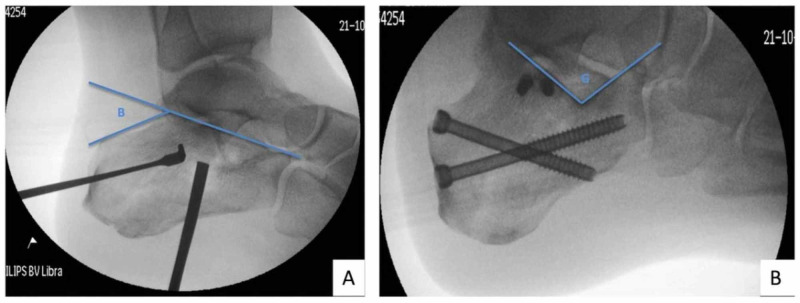
(A) Fluoroscopy-guided reduction of displaced posterior facet and restoration of Bohler’s angle (B), as shown by blue lines. (B) Final position of MIS fixation and Gissane’s angle (G), as shown by blue lines. MIS, minimally invasive surgery

Both Bohler’s and Gissane’s angles were measured on lateral radiograph using PACS. Bohler’s angle was measured by subtending two lines. The first line is drawn by connecting the highest points of anterior and posterior facets. The second line runs tangential to the superior edge of tuberosity (Figure 3A). An angle of 20 to 40 degrees is usually regarded as normal [5]. The Gissane’s angle is an obtuse angle formed by the downward and upward slopes of the superior surface of the calcaneum measured on the lateral border directly inferior to the lateral process of the talus (Figure 3B). Its normal value is between 120 and 145 degrees [5].

## Results

A total of 39 calcanea (37 patients) were operated, of which 20 had open procedure and 19 had MIS procedure, including one bilateral surgery in each group. Bilateral surgery was performed on the same day for both sides in both groups. Of the 37 patients, 29 were males and 8 were females. Mean age of the patients in the MIS group was 42.18 years (range: 15-68 years) and that of the patients in the open group was 43 years (range: 21-75 years). Of the fractures, 53.84% (n = 21) were Sanders type III, 28.20% (n = 11) were type II and 17.94% (n = 7) were type IV. Both groups had all three types of CFs (Table 1).

**Table 1:** Demographic data and types of calcaneal fracture according to Sanders classification *One patient in each group underwent bilateral surgery. MIS, minimally invasive surgery; ORIF, open reduction internal fixation

	MIS (n = 18*)	ORIF (n = 19*)	All patients (n = 37)
Mean age, years (range)	42.18 (15-68)	43 (21-75)	42.67 (15-75)
Sex			
Male	13	16	29
Female	5	3	8
Side			
Right	11	12	23
Left	8	8	16
Sanders type, n (percentage)			
Type II	3 (15.78%)	8 (40%)	11 (28.20%)
Type III	11 (57.89%)	10 (50%)	21 (53.84%)
Type IV	5 (26.31%)	2 (10%)	7 (17.94%)

In the MIS group, Bohler’s angle improved from a pre-operative mean of 7.7 degrees to 32.6 degrees and Gissane’s angle improved from a pre-operative mean of 152.7 degrees to 136.3 degrees. In the open group, Bohler’s angle improved from a pre-operative mean of 7.7 degrees to 31.5 degrees and Gissane’s angle improved from a pre-operative mean of 155.8 degrees to 139.2 degrees. Mean corrections are detailed in Table 2. Further analysis of the cohort showed that patients over 60 years of age (n = 7) had significantly narrower post-operative calcaneal width in the MIS group.

**Table 2:** Comparison of mean surgical outcomes MIS, minimally invasive surgery; ORIF, open reduction internal fixation

	MIS	ORIF	p-Value
Heel height correction (mm)	5.3	5.0	0.866
Heel width correction (mm)	-2.7	-2.3	0.803
Bohler’s angle correction	24.9	23.8	0.811
Post-operative Bohler’s angle	32.6	31.5	0.713
Gissane’s angle correction	-16.4	-16.6	0.954
Post-operative Gissane’s angle	136.3	139.2	0.142

All fixations showed radiological evidence of union by six months follow-up, which was confirmed by plain radiograph. Neither open nor minimally invasive group resulted in any post-operative wound infections in our series. Mean hospital stay for the MIS group was four nights (range: 2-13 nights) and that of the open group was five nights (range: 2-17 nights).

The mean cost for implant used for open procedure was £882.79, and the implant cost per patient for MIS was £142.89 (Table 3). The difference was significant. Mean utilisation of cumulative X-ray dose was significantly higher in MIS (0.764 mGy) in comparison to the same in open surgery (0.392 mGy), as shown in Table 4. The result was also statistically significant.

**Table 3:** Difference in cost of implant used in MIS and open calcaneal fracture surgery MIS, minimally invasive surgery; ORIF, open reduction internal fixation

	Cost of Implant (Pound Stirling)	
	MIS	ORIF
Mean	142.90	882.79
Min-Max	39.42-236.52	780.30-1174.50
Standard deviation	58.92	96.07
P-value	<0.001	

**Table 4:** Difference in utilisation of cumulative X-ray dose in MIS and ORIF MIS, minimally invasive surgery; ORIF, open reduction internal fixation

	Cumulative X-Ray Dose (in mGy)	
	MIS	ORIF
Mean	0.764	0.392
Standard deviation	0.53	0.16
P-value	0.027	

Average surgical time for MIS was 64.9 minutes and that of open surgery was 106.3 minutes. Average waiting time for MIS was 6.6 days and that for ORIF was 9.8 days. Wound healing was quicker (average 13.4 days) in MIS than ORIF (average 17.2 days). All these differences were statistically significant (Table 5).

**Table 5:** Surgical timings and wound healing comparison MIS, minimally invasive surgery; ORIF, open reduction internal fixation

	MIS		ORIF		p-Value
	Min-Max	Average	Min-Max	Average	
Surgical time (minutes)	33-90	64.9	65-145	106.3	<0.001
Time to surgery (days)	0-13	6.6	4-16	9.8	0.025
Wound healing time (days)	10-16	13.4	14-21	17.2	

## Discussion

Both operative and conservative treatment have been used for displaced intra-articular CFs. Various surgical techniques have been described. These include the lateral extensile “L” approach, MIS sinus tarsi approach, percutaneous approach, double external fixator distraction technique, combination approach, balloon-assisted calcaneoplasty, subtalar arthroscopy assisted fixation and arthrodesis [3,8-12]. No evidence for a gold standard treatment has been reported.

The distribution of cases in our study revealed that type II Sanders was about three times more in the ORIF group, whereas type IV Sanders was about 2.5 times more in the MIS group (Table 1). Further analysis of the cohort showed that most of the ORIFs (n = 18.90%) were performed by 2016 and only five MIS (around 26%) were performed during this period. In our centre, most of the calcaneal surgery after 2016 was performed following MIS technique. Interestingly, Griffin et al. published their report of a multicentre Randomised Control Trial, UK Heel Fracture Trial, in 2014 [13]. The finding of this trial has shown no symptomatic or functional advantage of operative outcome in comparison to non-operative treatment. It has also reported high risk of complication and re-operation following calcaneal surgery; hence, they recommended against it. It appears that in our centre, type II and type III Sanders fractures were treated by MIS as the technique got popularity over ORIF during this period.

The extensile lateral approach has reported good to excellent clinical outcome in 60-85% of cases [14]. Open surgery has been associated with high rates of wound complications. In some series, it has been up to 25%, where 21% of them required surgical treatment [2].

Wang et al. included 492 CFs and looked into the functional outcome of open and MIS procedure [1]. They did not find significant difference in outcome, which is supported by our radiological findings in both groups. Another similar comparative study by Weber et al. included 24 calcaneum operated by limited open reduction and 26 calcaneum was operated by conventional extensile lateral approach [12]. In this study, there was no wound complication in the MIS group and only four minor complications in open surgical group. Several other case series have shown positive outcome with MIS for displaced intra-articular calcaneum fracture where arthroscopic assisted reduction was performed [8-10].

In our cohort of open surgery, Bohler’s angle improved from a pre-operative mean of 7.7 degrees to 31.5 degrees (average correction: 23.8 degrees) and Gissane’s angle improved from a pre-operative mean of 155.8 degrees to 139.2 degrees (average correction: 16.6 degrees). We could not find any statistically significant difference irrespective of types of fracture as our patients were equally distributed in both groups. This result is comparable to the one published by Jain et al. where the mean Bohler’s angle improved from 4.15 degrees to 25.47 degrees and mean Gissane’s angle improved from 151 degrees to 121 degrees [14]. A small cohort (n = 7) of our patients over 60 years of age has been found to have a significant correction of calcaneal width after MIS (n = 4; average heel width correction of 4.5 mm) in comparison to ORIF (n = 3; average heel width correction of 2.3 mm). In over 60 years, MIS might be a better option than open surgery, although it will require a study with a larger number to reach a conclusion.

No study was found looking into radiation dose and wound healing time in CF surgery. Our study has compared those factors in both the ORIF and MIS groups. In our series, the open group required significantly longer (p < 0.001) time for wound healing in comparison to the MIS group (Table 5). This is due to the larger incision and more dissection required in extensile approach.

In our cohort of open surgery, Zimmer Biomet A.L.P.S. calcaneal plates with variable number (6-15) locking screws were used. In the MIS group, two to six cannulated screws were used. Price of each A.L.P.S. plate was £583.20 and that of each screw was £39.42. We found a significant difference (p < 0.001) in implant cost in those two groups. We have only taken the cost of implants into consideration, although inclusion of prolonged hospital stay, more instruments and theatre equipment will certainly make the difference even pronounced. In our series, patients in the open group stayed one extra night in hospital on an average. A study by Clement et al. has shown significantly higher costs in open procedure comparing to other treatment [15]. MIS was found to be the least expensive followed by conservative and open surgery.

Our study is the first study looking at radiation dosage in different modalities of CFs. We noticed utilisation of significantly higher (p = 0.027) fluoroscopic time and hence cumulative radiation dose in MIS. The mean cumulative radiation dose required for MIS was 0.764 mGy and that for and open procedure was 0.392 mGy. In other words, double radiation dose was required in MIS on an average. It is similar to being exposed to 2.5 more standard chest X-rays, which require a mean entrance surface dose of 0.15 mGy [16]. This is most likely due to the higher requirement of X-ray guidance for articular reduction with limited surgical exposure. This finding should remind us of the As Low As Reasonably Possible (ALARA) radiation safety principle [17]. It might be useful to bear in mind, regardless of procedure concerned, that the maximum annual dose limit is 20 mSv for the body, 150 mSv for thyroid and eyes and 900 mSv for the hands [18], especially when only 24% of surgical trainees are reported to use a thyroid shield [19].

We understand that our study population was small, and studies including a larger number of patients would be required to conclude whether one method is superior than the other. Another limitation of our study is not to use any patient-reported outcome measures, as our aim was to find out radiological outcome in the first place. Assessment of a wider range of clinical outcomes, e.g., using the Manchester-Oxford Foot Questionnaire, would be useful to find the overall clinical outcome. We recommend multicentred randomised controlled trials with a large number of populations for comparative study among MIS and non-operative management.

## Conclusions

Minimally invasive calcaneal surgery is cheaper and quicker and can safely be performed early. It is associated with early wound healing and can achieve equally good radiological correction. We recommend evaluating every case on its own merit and to consider MIS while deciding on the surgical management of CFs.
